# Drug Use and Spatial Dynamics of Household Allocation

**DOI:** 10.4172/2155-6105.1000275

**Published:** 2015-04-10

**Authors:** Eloise Dunlap, Emma J Brown

**Affiliations:** 1 National Development and Research Institute, New York, USA; 2 South Marion Avenue, Lake City, Florida, USA

**Keywords:** Drug use, Drug sales, Household space, Women and drug use, Behavior patterns, Socialization, Drug subculture

## Abstract

Household space allocation by women who consume drugs in New York and North Florida is depicted to demonstrate the complex character of household space and social relations. Some parents attempt to hide their drug consumption through the allocation space in the household for drug use. Women allocation of space for drug use within their households and the impact of this on the household are relevant issues with implications for therapy and prevention.

**Objective:**

The use of household space has not been a focus of social scientists. Middle class households have been used by decoration literature to specify space utilization. Modest literature pay attention to the utilization of household space among drug focused households. Analysis herein looks at the lived social relations of drug users to their children through controlling household space.

**Methods:**

Data presented comes from two studies, New York and Florida. The studies involved a total of 158 participants in 72 families from New York and 26 participants in 23 families in North Florida. Both researches used an ethnographic methodology focusing on a variety of behavior patterns and conduct norms occurring within drug abusing households. Repeated interviews and observations took place in households which were visited at different times and days of the week. Florida study was conducted over a 2-year period; New York study took place over a 5-year period.

**Results:**

Data suggest parents attempted to conceal their drug use from their offspring by using various strategies. Mental, social, and physical were tied together in space allocation. Household space acquired a different meaning and arose from use practice.

**Conclusion:**

In urban and rural settings a pattern of household allocation space and drug consumption is emerging. Although drug consumption is still prominent, it is not all consuming or the primary focus in the lives of women who use drugs. These women may have learned to integrate their consumption into their daily household/family life through the reallocation of space in their homes.

## Introduction

This paper draws examples from both rural and urban settings to demonstrate behavior patterns with regards to household space utilization for drug use. Household space allocation by women who consume drugs in inner city New York and rural North Florida is depicted to demonstrate the complex character of household space and social relations. Routine drug use setting and the decision making processes which determine particular venues are herein given special emphasis. Evidence indicates that some parents attempt to hide their drug consumption through the allocation of specific space in the household for drug consumption.

Understanding set and setting is very important when looking at the social context in which illicit drug consumption occurs [1]. Mothers and fathers drug consumption behavior patterns are particularly warranted given the influence that modeling of drug use may have on their children [2]. Some literatures stipulate children exposed to their parents’ and their parents’ friends’ drug consumption behavior may be more vulnerable to adopting a drug-related identity [3], which may increase the children’s risk of drug use in the future.

Other literatures have indicated that parents use various strategies to reduce the harm of their drug participation on their offspring [4,5]. Such factors are critical when attempting to understand why all children with drug dependent parents do not use drugs or participate in the drug subculture. The drug dependent parents’ drug use has differential impact upon their offspring. Findings from this study suggest parents’ allocation of space for drug consumption in the household out of the sight of their offspring and other non-drug using family members may prevent children from drug use. Women allocation of space for drug use within their households and the impact of this on the household are relevant issues with implications for prevention.

## Theoretical Tenets

The idea that space is meaningful is familiar to social scientists. This paper draws from critical theoretical concepts in order to examine household space allocation. Zinberg looked at the psychological and social (set and setting) context in which addictive behavior develops [1]. Gottdiener introduced Lefebvre’s theory from a sociological perspective concerning the production of space [6,7]. In Lefebvre’s concepts, the importance of space is to be understood according to its manifestations as perceived, conceived, and lived [7]. Space has a complex character and enters social relations at all levels: a physical environment that can be perceived; a semiotic abstraction that informs how ordinary people negotiate space (the mental maps studied by geographers); social relations; and finally a medium through which the body lives out its life in interaction with other bodies [6]. Social relations and spatial relations are interconnected so that one cannot be examined without the other. Lefebvre ties together the physical, the mental and the social. He developed a generalized approach to space and introduced the idea that space is simultaneously “a special practice (externalized, material environment), a representation of space (a conceptual model used to direct practice), and a space of representation (the lived social relation of users to the environment)” [7].

Lefebvre applied his framework to the analysis of different environments and combined geographical, historical, and semiotic analysis. He stressed various societies and how they have particularized space in form and meaning over time. For Lefebvre there is a distinction between abstract space and social space. Abstract relates to the intersection of knowledge and power. It is the everyday lived experiences that is externalized and “materialized through action by all members of society [7].

Generally the ordinary use of household space has not been a focus of social scientists. Ordinarily household space is addressed by home decoration literature which uses the middle class household to specify household space and the utilization of such. Very little if any literature pay attention to the utilization of household space among drug focused households. In a limited respect Lefebvre’s theory can guide such an analysis with philosophical tenets of social learning theory [2]; set and setting [1]; phenomenology [8,9]; and symbolic interactionism [10]. The unifying theme running through these perspectives is the importance of understanding the meanings of human behavior and the socio-cultural context in which interactions and specific arrangements occur.

Lefebvre’s ideas on social space and how this arises from practice is important in looking at the utilization and rearranging of household space [7]. Social space gives attention to the everyday lived experiences that is externalized and materialized through the actions of women in their households. Through this examination of drug households we look at the lived social relations of drug users to their children through controlling household space.

Phenomenology places emphasis upon the fundamental determinations of lived experiences [11]. The task of phenomenology is to make manifest what is hidden in ordinary everyday experience. The structure of everydayness is interconnected with social roles and purposes [12]. Household space is subjectively constructed. While household space allocation and what each room is to be used for is taken for granted by everyone (culturally constructed), its everyday meaning is structured over time subjectively attached to a variety of meanings to living space allocation. Phenomenology is basically concerned with the description of experience. It is a philosophical method restricted to careful analysis of the intellectual processes, which are introspectively described without making any assumptions about their supposed causal connections to existent external objects.

Phenomenology seeks to render audible and articulate that which is silent. For example, awareness of each room in a house/apartment, taking for granted how these rooms are used; kitchens are for cooking and so forth. Everyday household space utilization is so ordinary that one does not recognize how this space may be used to incorporate drug use and sales into normal household living space. Household lived experience through space allocation is understood. The understanding of family life lies in this experience. However lived experience goes beyond direct experience of meaning. What situates and makes possible household interaction patterns is space allocation which may be more than the meanings actually realized in the course of individual lived experience due to the ordinariness of the experience. Phenomenology then helps to carry us over from what is familiar in an everyday way to what lies hidden in that familiarity as its meaning and ground. The structure of everydayness is an interconnected system of social roles and purposes.

Symbolic interactionism emphasizes the subjective meaning of human behavior. In this perspective the focus is upon the subjective aspects of social life rather than the macro-structural aspects of social systems. The theoretical perspective is based on the image of humans rather than on society. People are actors who continually adjust their behavior to the actions of others. People can adjust their behavior/actions because they are able to interpret them. They are able to denote actions symbolically and treat those actions and those who perform them as symbolic objects. Individuals have the ability to rehearse alternative lines of action before acting. Individuals can think about and react to their own actions as symbolic objects. They are therefore active creative participants who construct their social world, and are not passive conforming objects of socialization.

The meaning of events requires attention to changeable and continually readjusting social processes. Through negotiation among people, temporary social constructed relations are formed which are in constant flux despite the relative stability in the basic framework governing those relations. Close contact and immersion in the everyday lives of the person is important for understanding the meaning of actions, definitions, and the process by which people construct the situation through their interaction and actions.

Blumer stipulated objects helps to identify people. In understanding how a person identified with or interprets an object, one can begin to understand the person [13-15]. For example, when mothers attempts to hide their drug use from their children, one surmises that mothers are not comfortable with their use and that their actions of space allocation is a mechanism by which they structure their drug use so that it will not harm their image as a parent. Their failure at successful hiding their use leads to intergenerational drug participation.

Understanding any social phenomenon requires unraveling the dynamic definitions and the interactional patterns of the social actors. In the social learning paradigm, a continuous reciprocal interaction occurs among personal factors, the immediate environment (e.g. family members and household activities), and the neighborhood. Individuals primarily learn by observation and talking so that people acquire large, integrated patterns of behavior without having to form them gradually by tedious trial and error [2]. Some complex behaviors can be produced only through the aid of modeling: “If children had no opportunity to hear the utterances of models, it would be virtually impossible to teach them the linguistic skills that constitute a language”.

## Method

Data presented comes from two ethnographic studies entitled “Co-Occurring Drug Use, Violence, and Behavior Patterns” (New York) and “An Ethnography: Drug Use among African American Women in Rural North Florida.” The studies involved a total of 158 participants in 72 families from inner city New York and 26 participants in 23 families in rural North Florida. All participants in both studies were drug users and or drug sellers. Both studies used an omnibus ethnographic methodology, which focused on a variety of behavior patterns and conduct norms that occurred. Extensive field notes were kept of observation. All interviews were audio taped to document and preserve the accuracy of responses. Respondents were given an Informed Consent and agreed to allow ethnographers to visit their household at many different times during the research. Repeated interviews and observations of the same individuals in their household at various times and days of the week were conducted over a 2-year period with the rural samples and a 5-year period with the urban sample.

The strategy of repeated visits at various times, days, months, years and direct observations was essential for validating the reality of drug use consumption patterns of household members. This method permitted documentation of the social contexts where focal behavior patterns were routinely evident. The social processes between participants and researchers through repeated visits and interviews over time is essential for understanding the reality of drug use and issues of space allocation for drug use consumption within households and communities.

## Findings

### Household consumption behavior patterns: Issues in household space

The following findings are from both inner city and rural respondents who used drugs; most were female and were the head of their households. Findings, in many instances, revealed that mothers attempted to conceal their drug use from their offspring by using various strategies. Rugrat (Florida) in talking about her mother’s drug use behavior related: “my mother smoke, but my mother never smoked in front of us. She used to always go in her room and open a window. We be asking her Ma, what was in there? ‘What y’all want?’ But she respected us, and that’s how we was raised. That’s why I can never say my mother influenced me.”

Rugrat’s mother demonstrated the mental, social, and physical tied together in space allocation. She designated her bedroom as special space and locked the door so that the children could not enter. This is interpreted as lived social relations of a drug user to her children. Her bedroom was particularized. This special space acquired a different meaning and arose from her use practice. The present findings are consistent with other literatures which found that women used a number of strategies to try to shield their children from the pitfalls of the drug subculture [5].

Many parents set aside special space in their homes where their drug use was out of the sight of non-drug using family members, adults as well as children. Others chose to use drugs away from their home when children were present or used drugs in their home only when their children were absent. Findings show that parents attempted to conceal their consumption from their children however the more intense drug consumption became, the less likely they attempted to hide their use. In such instances, consumption occurred more often outside of the household.

### Personal “Set Aside” household space

Respondents’ experiences give a picture of how parents, particularly mothers attempted to shield their children from their drug use and how they employed household space to do this.

Similar to Rugrat, Brown Eyes (New York) revealed how her mother attempted to set aside private space for consumption in the household. Brown Eyes, one of four children, parents were heavily involved in drug/street subculture. Both parents sold and used crack. Her father was arrested and deported back to his hometown when she was approximately 13 years old. This left her mother, Skins, alone to care for four children. At the time when Brown Eyes talked of her experiences, she was 16 years old. Before and after her father was deported both parents consumed drugs in the household. Generally, the parents when alone used their bedroom for consumption. When a limited amount of friends visited, they took their company into their bedroom with them. However, when a number of “friends” visited (which was often due to drug distribution activities) the children were relegated to their small bedroom and not permitted to interact with parents or their company. At this time the living room and kitchen became inaccessible to the children. They were expected to remain in their room with little interaction with the parents.

This is exemplified in Phe (Florida), through talking about her growing up years and parents drug use

### Phe

“When I was little I was scared of my mother, she was always hitting me, and she was always slapping me. The kitchen was where she used to get high. This lady, her boyfriend and her two kids use to stay there. And there use to be a lot of crack heads in and out the house all the time. If I was to come to the kitchen tell my mother I’m hungry she would get up and slap me. Cause I’m seeing something -- Cause they use to be getting high in the kitchen.”

The mother did not want the children to see drug use; they were not allowed to enter into the space that had been carved out for drug use. As seen when the mother slapped Phe, she did so because Phe was “seeing something” that she was not to see.

Carmen (New York), unlike Rugrat, Brown Eyes and Phe, was totally unaware of her parents’ drug participation. Her mother and step father were partially successful in concealing their drug use to their children through the allocation of household space.

“The only reason I know that my stepfather used is I remember seeing him have a heroin withdrawal, no, almost an overdose. Well, his heroin use didn’t really affect me, besides that time I seen him almost go into cardiac arrest or whatever, shaking on the floor. My mother told me that he was having a heart attack. But now that I think back, they threw a vial down the toilet. I remember him waking up so fast, and I remember the ambulance people saying, I saved your life. And then they go into the toilet and flush it down the toilet. So now I put two and two together; it wasn’t no heart attack, because he was all, you know; and they didn’t take him to the hospital. They just gave him a shot; and then he just got up like nothing was ever wrong.”

In talking about her mother and her growing up years Carmen related that she did not know that her mother used drugs. In relating her experiences in growing up it is clear that Carmen’s parents used drugs in the household but were successful in concealing such through the allocation of household space. The stepfather having an attack in the household indicates they had used in the house but out of the sight of the children. Later, in talking about her mother on methadone, Carmen related that she had never known her mother to use heroin but realized that she must have been a user all the time because there was no other reason for her to be on methadone. She was unsure when her mother began to use heroin or when she stopped because she had never seen her “shoot up” or “snort” heroin. Implied here is that the parents were mindful of using in the presence of the children.

Whitney (New York) revealed the importance of the bathroom as a set aside household space for her mother’s drug consumption:

“No, I never saw her do it. She would go in the bathroom most of the time. And how I know that is because when she would go a lot of times she left blood on the sink, you know, because she would shoot up. And she would leave blood on the sink, or whatever. Up until about the age of ten, I think I really didn’t know she was using drugs.”

Here we have Whitney’s mother designating the bathroom as her private space. In addition, Whitney also reveals that she was unaware of her mother’s use until she was ten years old. As a child she wondered about the blood on the sink. For the early years of her children’s growing up years, Whitney’s mother was able to conceal her use from her children. The following field notes elaborate further the use of the bathroom as household space set aside for drug use.

Rhonda and JT (New York) revealed attempts at normalizing drug consumption into the daily routine of their personal and family life. These are grandparents whose grand children lived between the grandmother and their mother:

“Today I decided to drop by to see Rhonda since I was in the area. I called Rhonda and she agreed that it would be OK for me to come over. Upon my arrival Rhonda answered the door. She related that JT was home and that I could interview him if I wanted to do so. When I entered the apartment Rhonda told me that J.T. was in the bathroom. She told me that he would be with me in a minute. I suspected that he was in the bathroom shooting his drugs. Rhonda confirmed my suspicion by saying, “You know he had to take care of business first.” When J.T. came out of the bathroom he appeared to be high. (Field notes).

Rhonda and JT have been observed numerous times using the bathroom as their private space for consumption. At the same time, she has been observed cleaning house and taking care of her grandchildren. A private space was designated for drug use or drug use occurred when the children were either asleep or away from home. Within this family interaction, the mental, physical and social come together in space allocation. Rhonda set aside a space for herself and JT to use their drugs while she also performed her role as a mother and grandmother being home much of the time.

These same factors are present in Shorty (New York) and her relations with her children although the household space allocation is radically different from that of Rhonda and her children. The unifying element here is women displaying specific behaviors in order to relay an important message to their children. The communication sent implies that they are fulfilling their parental role and are still functional in their role as head of their household. Observations revealed Shorty also set aside special space in the household where she smoked crack. She too maintained her household chores and kept her drug use separate from her household affairs. Shorty was often observed cleaning house and cooking for her children. The following field notes revealed observations of Shorty’s space allocation:

“When Shorty returned (from opening the door) she was followed by a woman and a man who both spoke to me. Shorty did not bother to introduce me to the male nor female but rather Shorty and the woman went into her bedroom, closing the door behind them while the man sat down on the sofa where I was sitting and began to stare at the television …Shorty’s brother, Jim, came into the living room and sat down . … It seemed like a long time, but probably ten minutes had passed before Shorty came out. When the door opened an odor emitted from the room, it was strong and I am sure it was the smell of crack. Shorty was noticeably different; she was more talkative and moved around more than she had moved previously, she was no longer sluggish as when I first saw her earlier today.

The room door was still open and the woman remained inside the bedroom. The woman yelled to the man (whose name I now learn is Harvey) to come into the room . … I realized that this was probably Shorty’s major source of drugs, people coming in and out of her home either to live or to use her apartment as a crack spot. Shorty was in and out of the room. Jim eventually went into the bedroom also. Finally the woman and Harvey came out. As they left Shorty walked them to the door. Jim was still in the bedroom. (Field notes New York).

Although Shorty had 3 children she turned her apartment into a crack consumption spot where individuals came to smoke. They shared what crack they had with her. She still attempted however, to control the situation by having consumers use her bedroom for smoking. It was the one room in the house in which she sat aside for her and others smoking activities.

In this case example, Shorty trained the children to know who to permit into the apartment and who to keep out. Another day in the field notes highlights his point.

“Minutes later a female walked into the living room; she did not acknowledge me. She did not say hello to Shorty, she simply held out her hand and gave Shorty a very tiny package. Shorty was upset by her actions and told the female that she was inappropriate because she did not know who I was. Shorty told her that I could have been anyone sitting there, a social worker, or even the police. The girl apologized and they both walked towards Shorty’s bedroom, Shorty was still fussing about her actions. They closed the door and in a small span of time Shorty came out of the room. I asked Shorty to show me the crack; she went into her bra and pulled out something, which looked like a small piece of paper. It was a very tiny package, with one small rock in it. She told me that she was angry with her son for letting this particular female into the apartment. She told me that this female gets paranoid and thinks that police are on the roof looking into the apartment.” (Field notes New York).

On this particular day her children were home. Her oldest son, 16 years old, served as the one to answer the door. Her younger son, 9 years old, alternated between looking at cartoons and going outside to play. Her 15 years old daughter who was home when I arrived was on the telephone in the kitchen most of the time. Unlike Rhonda, Shorty involved her children in her drug activities. She trained them on what to do when users came to the household. They did not go into Shorty’s bedroom; they understood it was off limits to them. Although Shorty turned her apartment into a place for drug consumption, she set aside her bedroom for consumption space. Shorty attempted to stabilize her role as household head through household space allocation, cleaning and cooking for her children.

A number of the mothers either did not use drugs in their household when their children were present as in Suga’s household (Florida) or restricted their children to a certain room in the house when they used as in Deborah’s household (New York).

Suga stated: “Nobody can use drugs in my house when my little girl’s around. They can’t do all that cussing and stuff around my little girl. And I don’t like, you know, smoking. I would rather they smoke outside when they over here and stuff like that.”

Deborah on the other hand related she used drugs when her children were not at home or she relegated them to a designated space within the household. The use of these strategies was not always successful as she related:

“I do not play that [using drugs in my house] when my Kids are home, but if like my kids are not home, and it’ll be like we can chill out

[use the house]. You know, like that, but it’s like, if they’re home, then like we going to stay out [not go to the house]. No actually, one time, my son didn’t see me, but it was like a couple of years ago and my son, we were like, he was home. He was the only one home. So I was like okay he can just go in the room with the TV. So he was in the room with the TV, but he happened to come out and was like “Wait a minute they is grown folks in here” and I say, “Go back in the room.” And he say “Mom, what you all gonna do? Put that stuff up your nose?” And I was like, where you getting that from?”

Similar to Deborah, Bobbie (New York) either smoked outside of her apartment or restricted her children to one room. She has four children ages 17, 16, 11 and 8. Bobbie generally stood in the hallway of her building to smoke crack. When she smoked in the hall, she stood on the fourth floor so that her children would not see her using; she lived on the fifth floor. Emphasis was placed upon relegating household space is again displayed. When Bobbie smoked in the house, she relegated her children to the back bedroom. Whenever she has company, all her children have to go into the one small bedroom in the back of the apartment.

Unlike some of the other women who relegated consumption in only one room, Bobbie required her children to remain in one room. When her friends stopped by her apartment and wanted to smoke, they did so in the living room. Since the living room was open and children could observe her use, she did not allow them to come out of their bedroom. As with the other parents, she too did not want her children to see her actually consume drugs and thus allocated household space.

## Non Set Aside Household Space

In contrast, Cookie (New York) had older siblings who used drugs in the household. Once their mother died, their apartment became a place where individuals came to shoot heroin and use other drugs.

Cookie: “But my sister and my brother that was using the drugs, they had their stuff on the table. And I didn’t know what it was. And so they had candles and stuff. So and they had a couple of other friends that were there. But I didn’t know what it was, so I went to the table, and I was like, ooh, a top-I used to play skelly, so I thought it was a skelly top. But it had the water and the cotton in it, or whatever; so I ain’t know what was going on. And then I saw the needles. And I was like, gosh, somebody was playing doctor. I’m a kid. I picked it up. And my sister nearly smacked me outta the door. And she said, “I never want to see you do this.” And so I got up and went in the bathroom. And now the lights were out, and I didn’t know someone was in the bathroom. And when I opened the bathroom, the way the bathroom was situated was, when you walked in the bathroom, you saw the sink first. And the toilet was the other way. So if you’re just going to the sink, you’re not gonna turn around and think to look behind you. And I saw this person sitting on my toilet with the needle in their arm. And I just ran outside; because I ain’t know what was going on. I just ran outside.”

This is a non prominent example in which the entire household was dedicated to the use and consumption of drugs. The single parent that was the head of the household however had passed away. The older children were drug users. They allowed drug consumption to take place in any room in the house.

## Discussion

This paper examined places of consumption in households in which illicit drug use occurred. Data suggests that parents, in most cases mothers, have found ways to integrate their drug use in their homes without their children observing them. In their attempts to shield their children from their use, they designated specific spaces in their household for use of drugs. As such, the children are not allowed to enter into this private space in the household while drug use is taking place. As exemplified in Shorty’s family, while a considerable amount of traffic was observed in the apartment, her guests was allowed to smoke in only one room. She set aside this room for consumption in her household whether her children were home or not. Although she could smoke in any room of the house she would not do so nor would she permit her company to do so. Everyone was required to go into the bedroom to smoke. If the living room was full of guests, they had to wait their turn to go into the bedroom. Such behaviors implied that parents saw themselves as in control of their addiction because they: a) set aside household space for drug consumption; b) limited where smoking could be carried out and c) kept their children from directly observing drug consumption.

In other instances, children were relegated to one room when drug use was taking place. They were not allowed to exit the room without the parent’s permission. The findings indicate the importance of space to be understood according to its manifestations as perceived, conceived and lived. This finding maybe viewed in terms of symbolic interactionism where mothers who use drugs react as others to their drug use. As actors they construct alternative modes of behavior with respect to their drug use and objectify themselves and may not want their children to see them as such. Therefore, they adjust their actions because they interpret their drug use as if they were the child and thus do not want the children to see what is taking place.

These findings indicate space as a complex character and enters social relations at all levels. It is both a physical environment that can be perceived and an abstraction that can inform us how people who use illicit drugs negotiate household space to consume in privacy from children and other family members. Generally, parents used various strategies to reduce the harm of their drug participation on their offspring. As Rugrat replied his drug use was not attributed to his mother because he never saw her use drugs, he felt that his mother respected them while he was growing up, she would lock the door to her bedroom and open the windows when she smoked her drug.

On the other hand, some parents were not successful in the allocation of house space for drug use or using when the children were absence from the home. For example, one parent was unsuccessful in trying to restrict her son to the one room to look at TV so she resorted to using drugs outside of the household or when the children were not at home.

Such factors are critical when attempting to understand why all children with drug dependent parents do not use drugs or participate in the drug subculture. Dunlap examining the households of women who consumed crack found that consumption behavior was not passively observed. It was not a passive receptive process. There was a great deal of emotional and psychological trauma that took place [13]. Children while growing up and observing their mother consume crack, upon becoming teenagers they too began to use drugs. They however, most often chose another drug of choice i.e. blunts [14].

This manuscript indicates within both urban and rural settings, the beginning of a pattern of household drug consumption is emerging. Although drug consumption is still prominent, in many cases it may not be all consuming as in the early years of drug consumption. It is not the only focus in the lives of women who use drugs. These women may have learned to integrate their consumption into their daily household/family and community life and still have the feeling that the children respected them. Thus the importance of space is to be understood according to its manifestation as perceived and lived in households in which drug use occurs.

When looking at the utilization of household space, findings indicate it has gone beyond traditional use, i.e. kitchen for cooking and eating, findings herein indicate further that space has a complex character and enters social relations between the parent and child at all levels. Traditionally, household space for consumption was particularized in form and meaning generally through defining how each room is to be used through magazines, movies, books, etc. These findings add another dimension to a look at household space through looking at drug users’ utilization of specific spaces in the household for drug use. Parents designated household space for consumption even though in some instances the space may be open without doors and in other cases the space may have doors for privacy. In the majority of cases, the children are expected not to enter such space when drug use is occurring.

There were instances in which mothers allowed incoming friends to bring drugs to be consumed but also restricted the space in the home where this could occur. These findings began to unveil the importance of drug users’ consumption and the socio-cultural (Street/Drug Subculture) context in which consumption and household arrangements occur. In these cases, the strategies appeared to be used to assure the mothers themselves and their children they were in control of their drug consumption and that the habit was not all encompassing. In doing so, women depicted a change in attitude, the readjustment of behavior and reallocation of household space as a social process. More studies need to examine household space within context of drugs/street subculture. Such information is critical for drug treatment and rehabilitation.

Also evident was an example where the entire household space was relegated to drug use and sales as seen through Cookie after the death of the mother. Generally, when the parent lost control, use of drugs throughout the home occurred, children were then eventually removed from the household and placed in foster care.

## Conclusion

In this paper we see how mothers interpret their drug use and readjust/allocate household space to accommodate it while at the same time seeing their drug use as something undesirable for their children to observe and emulate. Within urban and rural settings, the beginning of a pattern of household drug consumption is emerging. Although, crack and other drug consumption is still prominent, in many cases it is not all consuming; it is not the only focus in the lives of women who use drugs. These women may have learned to integrate their consumption into their daily household/family and community life.
